# Family Medicine and Primary Healthcare: The Past, Present and Future

**DOI:** 10.3390/healthcare11152128

**Published:** 2023-07-26

**Authors:** Antonella Arghittu, Paolo Castiglia, Marco Dettori

**Affiliations:** 1 Department of Medicine, Surgery and Pharmacy, University of Sassari, 07100 Sassari, Italy; aarghittu@uniss.it (A.A.); castigli@uniss.it (P.C.); 2 University Hospital of Sassari, 07100 Sassari, Italy; 3 Department of Restorative, Pediatric and Preventive Dentistry, University of Bern, 3012 Bern, Switzerland

## 1. Background

As defined by the World Health Organisation in the conference held in Alma Ata, Kazakhstan, in 1978, “Primary health care is essential health care based on practical, scientifically sound, and socially acceptable methods and technology made universally accessible to individuals and families in the community through their full participation and at a cost that the community and country can afford to maintain at every stage of their development in the spirit of self-reliance and self-determination” [1]. This definition marked a turning point in the concept of primary care, arguing for a shift from the traditional biomedical (disease-centred) model, based on the treatment of single episodes of illness, to a bio-psycho-social (patient-centred) model centred on prevention, continuity of care, health communication and the direct role of patients in disease management [2,3,4]. From this perspective, the “patient-centred” model does not exhaust the role of the health professional, nor does it negate the “disease-centred” model, but rather combines the traditional pathway of diagnosis and treatment according to evidence-based medicine (EBM) with the anthropological and subjective meaning offered by the patient’s perception of him/herself [5,6,7].

More than forty years after Alma Ata, this proposal continues to respond effectively to the challenge posed by epidemiological changes (the prevalence of chronic diseases), social changes (growth in health inequalities) and cultural changes (patients’ demand for more information and autonomy) in an ever-evolving society, in which family medicine acts as a link between local and hospital health settings, patients and specialists and prevention and treatment. [8,9,10].

In this context, the digital revolution heralded by Information and Communication Technology (ICT) has also changed the doctor–patient relationship. Indeed, today’s society enjoys advanced technologies related to artificial intelligence, cloud computing or IoT (Internet of Things); efficient information systems; and e-health, which are now considered promising tools and strategies to promote health and well-being for the whole population [11,12,13]. 

In particular, mobile health (m-health), defined as a public health practice supported by mobile devices, was widely used to ease the health burden during the COVID-19 pandemic [14,15]. In this regard, m-health tools have been used by General Practitioners (GPs) to monitor lifestyles adopted during isolation (i.e., monitoring physical activity and proper nutrition), to remotely guide self-medication practices wherever and whenever necessary by recording disease parameters, to monitor adherence to prescribed drug therapies and to share information and reports with patients and health professionals, thereby helping to support primary care at a time of high demand for care [14,15,16,17]. 

Nevertheless, while new digital systems have enabled citizens to self-manage, they have also affected the deviation of perceived risk from real risk, sometimes leading to inappropriate behaviour in both hospital and community settings [18,19,20,21,22,23]. Here, again, the role of primary care medicine, with general medicine, the continuity of care service and paediatrics at the forefront, has been decisive in guiding patients and citizens towards the adoption of correct behaviour and health choices. An example in point is the commitment to vaccine counselling at a time when the determinants of vaccine hesitancy have been exacerbated by the emergence of a newly synthesised vaccine (anti-SARS-CoV-2) or the workload assumed in order to cope with complex situations in extremely precarious conditions from an organisational point of view [24,25]. These factors are among the main causes of the transformation of the care needs to which the social and health services need to respond.

Today, family medicine is an academic and scientific discipline, with its own educational and research content, its own evidence base and a clinical speciality oriented towards primary care [26,27]. 

Addressing a target group of patients differentiated according to macro-areas of clinical and care needs and represented in the different phases of life (childhood, adulthood and old age), family medicine constitutes the most inclusive, fair and efficient approach to improve the physical and mental health of individuals, as well as the well-being of society from a One Health perspective [1,2,3,4,27]. 

The characteristics of patients, in fact, are extremely diverse and vary in relation to the different types of needs and interventions. On the one hand, healthy (or apparently healthy) individuals require proactive and personalised preventive action that includes the promotion of healthy lifestyles together with adherence to vaccination prophylaxis and cancer screening [28,29,30,31]. On the other hand, individuals with new-onset diseases require the guarantee of a timely response accompanied by ad hoc counselling, ranging from simple health information to direct management for basic problems up to the referral to specialist services [32,33]. 

In addition, the number of individuals with chronic diseases is steadily increasing, with a high prevalence (overweight, hypertension, dyslipidaemia, diabetes, COPD, etc.) who need to be taken care of on a continuous basis over time and during transitions between the various levels of care; lastly, there are individuals in fragile conditions (physical, mental, social or economic) and/or with compromised personal autonomy and/or with complex care needs requiring the coordination and cooperation of all the various players indicated for the specific situation [34,35,36].

Today, ongoing demographic and epidemiological transformations are revealing that the population is ageing substantially, with the consequent increase in the prevalence of chronic-degenerative pathologies, the widening of the social inequality gap and the increase in the variability in community health needs, which are aspects directly linked to the processes of globalisation [37]. Moreover, the global burden of disease has changed a great deal over the last thirty years, increasing the need to focus on the organisation of health services. In such a scenario, it is becoming increasingly evident that the hospital setting cannot only represent effective, efficient and appropriate responses to often very complex frameworks that, on the contrary, require multidisciplinary and inter-sectoral care in the medium to long term [38,39,40,41,42,43].

By focusing on the health of the entire community, family medicine, as the foundation of primary healthcare, thus involves aspects of the organisation of healthcare systems, with a conceptual model characterised by (i) the accessibility and equity of the services offered, (ii) the centrality of the population’s needs; (iii) the “cost-effective” and appropriate use of technologies and services, (iv) the integration of the different levels of care (primary, secondary and tertiary) and (v) the involvement of the patient and the community in the care project [44,45].

However, despite the recognised importance of general practice in all areas of healthcare, a growing crisis threatens the stability and effectiveness of this vital component of the healthcare system, namely the notable shortage of General Practitioners. This shortage has serious implications for patient care and the overall health of communities. Consider that in 2020, the pivotal year of a pandemic that claimed millions of lives worldwide in the absence of a vaccine, only 20% of physicians in EU countries were GPs [46]. In this context, health promotion is called upon to characterise health policies not only with the objective of preventing the onset of pathological conditions but also to create a level of competence and capacity for control (empowerment) that maintains or improves health capital in the community. 

This means it is increasingly urgent to strengthen and innovate the network of territorial social-health services and to devise new integration strategies, promoting the active participation of citizenship [47].

A Special Issue entitled “The 10th Anniversary of Healthcare—Family Medicine” was launched in *Healthcare* in December 2022 and is a collection of original data or international literature on the role played by family medicine in protecting and promoting the population’s health. This Special Issue aims to publish scientific evidence on the following topics: primary care and continuity of care; new challenges in relation to emerging diseases; vaccination and screening; prescriptive appropriateness; combating antimicrobial resistance (AMR) and antimicrobial stewardship.

## 2. Manuscripts Published in the Special Issue

As evidence of the interest generated by the aforementioned topics of debate, as of 10 July 2023, 18 manuscripts have been accepted for publication in the Special Issue entitled “The 10th Anniversary of Healthcare—Family Medicine” following the peer review process. Specifically, 2 reviews, 1 perspective and 15 articles were published online [39,40,48,49,50,51,52,53,54,55,56,57,58,59,60,61,62,63]. 

Several topics are addressed, and all manuscripts are published and available online in open-access form. They are listed in chronological order of publication in Table 1, along with the following information: the authorship (first author), topic, time of investigation, methodology, and main results of the study.

## 3. Discussion

Numerous topics are addressed in the Special Issue entitled “The 10th Anniversary of Healthcare—Family Medicine”. Some of the studies included investigated the knowledge, attitudes and behaviour of primary care physicians in relation to various aspects: practices of evidence-based medicine (EBM) among primary care physicians in Saudi Arabia [52]; the diagnostic and therapeutic management of *Helicobacter pylori* infection [56]; and pharmaceutical prescription errors in intensive care units [59] and palliative care education and training in Malaysia [60], particularly exploring the importance of the academic training of university students regarding the need to request diagnostic investigations in order to develop a reflexive approach regarding prescriptive appropriateness [49]. 

The role of clinical trials in the context of family medicine was examined in relation to the attitude of the general population towards compliant participation in studies of this type, aimed at improving public awareness of the importance of participating in clinical trials necessary for the development of new drugs [61]. Of particular interest were aspects related to the importance of family medicine in the management of psychological and emotional disorders; in particular, the following were examined: (i) the relationship between family background associated with Attention Deficit Hyperactivity Disorder (ADHD) symptoms [48] and mental health problems among students attending the psychological support clinic at their university [54], (ii) the mediating role that psychological resilience plays in the relationship between family health and health literacy among university students [63], (iii) the relationship between depressive symptoms and socio-demographic factors among fertile Jordanian and Palestinian couples [50], (iv) the possible relationship between depressive disorders and clinic (glycated haemoglobin levels) in elderly patients with diabetes [40] and (v) the burden of caregivers of people with dementia through stress detection instruments [53].

Two of the articles published in the Special Issue examined the impact of the COVID-19 pandemic on primary healthcare with regard to the support of domestic violence cases during the lockdown in Mexico [57] and the working conditions of primary care physicians who were faced with great difficulties in managing patients, whereby remote care was added to routine care, adding to the already overwhelming burden of primary care with limited human resources [55]. Other topics, such as the study of the relationship between sarcopenic obesity and auditory function [51], the administration of useful drugs to reduce weight in obese patients [58], the evaluation of the clinical management of specific dermatological conditions by primary care physicians [39] and the description of the shortage of GPs in relation to the need to enhance university training in primary healthcare [62], were also addressed in this Special Issue.

This Special Issue, “The 10th Anniversary of Healthcare—Family Medicine”, aims to identify further best practices of proven effectiveness in the field for the implementation of problem-solving strategies to achieve health goals. Articles, systematic reviews, meta-analyses, short communications, short reports, letters or other types of articles dealing with the multidisciplinary nature of these topics and their impact on public health, also with reference to different population cohorts (infants, adolescents, adults, elderly and at-risk populations), are welcomed and encouraged to be submitted. Manuscripts may be submitted to this Special Issue by 31 October 2023.

## Figures and Tables

**Table 1:** Description of the manuscripts accepted for the Special Issue “The 10th Anniversary of Healthcare—Family Medicine” in chronological publication order.

Authorship	Research Topic	Time Frame	Methodology	Main Findings
Choksomngam, Y. et al. [48]	To find the association between each dimension of family functioning and controlled Attention Deficit Hyperactivity Disorder (ADHD) symptoms	2017–2018	Cross-sectional study	Family functioning was associated with controlled ADHD symptoms. In children with ADHD with poor symptom control, the assessment of family functioning could be helpful for children and caregivers.
Herbaux, C. et al. [49]	To improve the training of medical students in the choice of diagnostic testing faced with a clinical case of general practice	2017	Prospective study	Academic training about the prescription of diagnostic testing in family medicine is essential to improve students’ level of conscientiousness and develop a reflective approach regarding prescriptive appropriateness.
Jaber, D. et al. [50]	To compare the national prevalence of depression and anxiety signs/symptoms and well as identify associated socio-demographic factors among fertile Jordanian and Palestinian couples	2018–2019	Cross-sectional study	Jordanian and Palestinian couples have a high burden of anxiety and depression symptoms. Health professionals and educators should focus on creating preventive procedures using evidence-based guidelines to support the mental health of Jordanian and Palestinian couples.
Sirirak, T. et al. [40]	To investigate the longitudinal association between depression and glycated haemoglobin (HbA1c) levels among elderly type 2 diabetic patients	2020–2021	Longitudinal study	Depression is an important factor in glycaemic control in type 2 diabetic older patients; thus, it is important to routinely screen mental health evaluation in type 2 diabetic older patients in every OPD visit, and a treatment plan or preventive program should be provided as soon as possible.
Park, C. et al. [51]	To investigate the association of Low Muscle Mass (LMM) and obesity on hearing loss in the general population	2012–2017	Cross-sectional study	There is a possible link between sarcopenic obesity and the development of hearing loss; therefore, further research is needed in order to demonstrate that targeted treatments for sarcopenic obesity can improve hearing function.
ALruwaili, B. et al. [52]	To assess the knowledge, attitudes and practices of evidence-based medicine (EBM) among primary care physicians in the provinces of northern Saudi Arabia	2022	Cross-sectional survey	Given the low knowledge and use of EBM applied to family medicine, appropriate training programs for primary care physicians are needed to improve knowledge of EBM and its importance in clinical practice.
Tokovska, M. et al. [53]	To investigate the burden of caregivers of people with dementia in Slovakia through a stress measurement tool (Relatives’ Stress Scale—RSS)	2019–2020	Online survey	Expanding the network of social support groups, operating a counselling hotline/chat and introducing national educational programs for close relatives of dementia patients are necessary interventions in order to improve caregivers’ quality of life and quality of care.
Luvira, V. et al. [54]	To determine associations between family background and mental health problems in a cohort of students who attended the mental health counselling clinic for college students	2018	Cross-sectional descriptive study	Family contexts, such as parental migration, unfamiliarity with parents, financial issues, family conflicts, communication failures in the family, the infidelity of parents and domestic violence, were associated with mental health problems in university students.
Schrimpf, A. et al. [55]	To examine the impact of the pandemic, especially the double burden of providing healthcare to patients with COVID-19/long COVID and routine care, on GPs’ work routines	2021–2022	Online survey	The COVID-19 pandemic continues to worsen the working conditions of primary care physicians and affect essential health services; therefore, urgent interventions need to be made by institutions in order to support primary healthcare in the management of patients in times of high demand.
Frèche, B. et al. [56]	To synthesise knowledge about the diagnostic and therapeutic management of Helicobacter pylori (Hp) infection in general practice	1982–2018	Narrative review	General Practitioners represent the first line of care in the management of Hp infection; thus, simplifying the recommendations available to GPs in their daily practice would optimise the identification of patients at risk and the management of Hp infection.
Rivera Rivera, L. et al. [57]	To measure the prevalence of cases of domestic violence against women and some associated factors during the COVID-19 pandemic in Mexico	2020	Online survey	From a prevention perspective, sociocultural, psychological, and ecological interventions are necessary. Further, such studies are needed to measure the phenomenon’s evolution after the pandemic and determine the impact of measures put in place to prevent it.
Kosmalski, M. et al. [58]	To summarise the latest evidence about weight-reducing drugs and the treatment of obesity, taking into account their effectiveness and the individualisation of use	2022	Review	Due to the increasing number of drugs that reduce body weight, therapeutic choice is increasingly difficult. Taking into account the increased risk for diabetic patients, performing diagnostic tests before taking these drugs is essential.
Giannetta, N. et al. [59]	To translate and validate a questionnaire regarding the use of KAB theory in the study of medication administration errors in ICUs from English into Persian	2019	Cross-sectional pilot study	The KAB medication administration errors in the ICU questionnaire mean it is a reliable and valid instrument to be used within Persian healthcare settings.
Hamdan, N. et al. [60]	To investigate the knowledge and attitudes of primary care physicians regarding palliative care in Malaysia	2020	Cross-sectional study	Physicians’ basic knowledge of palliative care knowledge in Malaysia is still inadequate. More concerted efforts need to be applied to ensure that palliative care training is integrated into educational programs.
Abouelkhei, M. et al. [61]	To assess the knowledge of and attitude towards clinical trials among the general population of northern KSA and to identify the factors associated with poor knowledge and attitudes among them	2021–2022	Cross-sectional study	Improving public awareness of the importance of clinical trial participation (necessary for new drug development) is an achievable goal through targeted health education programs
Alotaibi, H. et al. [39]	To assess how dermatological diseases are identified, managed and referred to in primary healthcare centres (PHCs)	2021–2022	Mixed methods study	Targeted training, the provision of seminars, and improvements in medical school curricula are critical with regard to quality of care performance in the clinical management of commonly treated dermatologic conditions by primary care physicians.
Wiedermann, C.J. [62]	To describe the problem of the shortage of primary care physicians by focusing on modernising medical curricula, creating new medical schools and implementing policy innovations	2023	Descriptive study	The growing medical shortage crisis in primary care is a significant threat to the stability and effectiveness of healthcare systems. Addressing this problem requires a multi-pronged approach encompassing various aspects, including medical education and collaboration between institutions.
Wang, Y.-Y. et al. [63]	To examine the relationship between family health and college students’ health literacy and the mediating role that psychological resilience plays in this relationship	2022	Survey	Family health may be a potential key factor in improving college students’ health literacy, whereas psychological resilience functions as a mediating mechanism.

## References

[1] Declaration of Alma-Ata. Proceedings of the International Conference on Primary Health Care.

[2] World Health Organization (2008). The World Health Report 2008: Primary Health Care, Now More than Ever.

[3] Hanson K., Brikci N., Erlangga D., Alebachew A., De Allegri M., Balabanova D., Wurie H. (2022). The Lancet Global Health Commission on Financing Primary Health Care: Putting People at the Center. Lancet Glob. Health.

[4] Macinko J., Starfield B., Shi L. (2003). The Contribution of Primary Care Systems to Health Outcomes within Organization for Economic Cooperation and Development (OECD) Countries, 1970–1998. Health Serv. Res..

[5] Dettori M., Castiglia P. (2022). COVID-19 and Digital Health: Evolution, Perspectives and Opportunities. Int. J. Environ. Res. Public Health.

[6] Llorente C., Revuelta G., Carrió M., Porta M. (2019). Scientists’ opinions and attitudes towards citizens’ understanding of science and their role in public engagement activities. PLoS ONE.

[7] Bravo P., Edwards A., Barr P.J., Scholl I., Elwyn G., McAllister M. (2015). Conceptualising patient empowerment: A mixed methods study. BMC Health Serv. Res..

[8] Starfield B. (2011). The hidden inequity in health care. Int. J. Equity Health.

[9] Campus G., Cocco F., Strohmenger L., Wolf T.G., Balian A., Arghittu A., Cagetti M.G. (2022). Inequalities in caries among pre-school Italian children with different background. BMC Pediatr..

[10] Arghittu A., Dettori M., Masia M.D., Azara A., Dempsey E., Castiglia P. (2019). Social deprivation indexes and anti-influenza vaccination coverage in the elderly in Sardinia, Italy, with a focus on the Sassari municipality. J. Prev. Med. Hyg..

[11] Arghittu A., Balletto G., Dettori M. (2023). Smart City and Well-Being: Opinions by the Guest Editors. Urban Sci..

[12] Nahavandi S. (2019). Industry 5.0—A Human-Centric Solution. Sustainability.

[13] Arghittu A., Deiana G., Dettori M., Dempsey E., Masia M.D., Palmieri A., Spano A.L., Azara A., Castiglia P. (2021). Web-based analysis on the role of Digital Media in Health Communication: The experience of VaccinarSinSardegna Website. Acta Biomed..

[14] Arghittu A., Dettori M., Dempsey E., Deiana G., Angelini C., Bechini A., Bertoni C., Boccalini S., Bonanni P., Cinquetti S. (2021). Health Communication in COVID-19 Era: Experiences from the Italian VaccinarSì Network Websites. Int. J. Environ. Res. Public Health..

[15] Mateo G.F., Granado-Font E., Ferré-Grau C., Montaña-Carreras X. (2015). Mobile Phone Apps to Promote Weight Loss and Increase Physical Activity: A Systematic Review and Meta-Analysis. J. Med. Internet Res..

[16] Rzymski P., Borkowski L., Drąg M., Flisiak R., Jemielity J., Krajewski J., Mastalerz-Migas A., Matyja A., Pyrć K., Simon K. (2021). The Strategies to Support the COVID-19 Vaccination with Evidence-Based Communication and Tackling Misinformation. Vaccines.

[17] Deiana G., Dettori M., Arghittu A., Azara A., Gabutti G., Castiglia P. (2023). Artificial Intelligence and Public Health: Evaluating ChatGPT Responses to Vaccination Myths and Misconceptions. Vaccines.

[18] World Health Organization Managing the COVID-19 Infodemic: Promoting Healthy Behaviours and Mitigating the Harm from Misinformation and Disinformation. https://www.who.int/news/item/23-09-2020-managing-the-covid-19-infodemic-promoting-healthy-behaviours-and-mitigating-the-harm-from-misinformation-and-disinformation.

[19] Masia M.D., Dettori M., Deriu M.G., Soddu S., Deriu M., Arghittu A., Azara A., Castiglia P. (2021). Microbial Monitoring as a Tool for Preventing Infectious Risk in the Operating Room: Results of 10 Years of Activity. Atmosphere.

[20] Deiana G., Arghittu A., Dettori M., Masia M.D., Deriu M.G., Piana A., Muroni M.R., Castiglia P., Azara A. (2021). Environmental Surveillance of *Legionella* spp. in an Italian University Hospital Results of 10 Years of Analysis. Water.

[21] Deiana G., Arghittu A., Dettori M., Deriu M.G., Palmieri A., Azara A., Castiglia P., Masia M.D. (2022). Ten-Year Evaluation of Thermal Comfort in Operating Rooms. Healthcare.

[22] Dettori M., Pittaluga P., Busonera G., Gugliotta C., Azara A., Piana A., Arghittu A., Castiglia P. (2020). Environmental Risks Perception among Citizens Living Near Industrial Plants: A Cross-Sectional Study. Int. J. Environ. Res. Public Health.

[23] Bonanni P., Angelillo I., Villani A., Biasci P., Scotti S., Russo R., Maio T., Rosati G.V., Barretta M., Bozzola E. (2021). Maintain and increase vaccination coverage in children, adolescents, adults and elderly people: Let’s avoid adding epidemics to the pandemic: Appeal from the Board of the Vaccination Calendar for Life in Italy: Main-tain and increase coverage also by re-organizing vaccination services and reassuring the population. Vaccine.

[24] Gostin L.O., Friedman E.A., Wetter S.A. (2020). Responding to COVID-19: How to Navigate a Public Health Emergency Legally and Ethically. Hastings Cent. Rep..

[25] Costantino C., Casuccio A., Restivo V. (2020). Vaccination and Vaccine Effectiveness: A Commentary of Special Issue Editors. Vaccines.

[26] Beasley J.W., Starfield B., van Weel C., Rosser W.W., Haq C.L. (2007). Global Health and Primary Care Research. J. Am. Board Fam. Med..

[27] Webster F., Krueger P., MacDonald H., Archibald D., Telner D., Bytautas J., Whitehead C. (2015). A scoping review of medical education research in family medicine. BMC Med. Educ..

[28] Grassi T., Bagordo F., Savio M., Rota M.C., Vitale F., Arghittu A., Sticchi L., Gabutti G. (2022). Sero-Epidemiological Study of *Bordetella pertussis* Infection in the Italian General Population. Vaccines.

[29] Dettori M., Arghittu A., Deiana G., Azara A., Masia M.D., Palmieri A., Spano A.L., Serra A., Castiglia P. (2021). Influenza Vaccination Strategies in Healthcare Workers: A Cohort Study (2018–2021) in an Italian University Hospital. Vaccines.

[30] Dettori M., Arghittu A., Castiglia P. (2022). Knowledge and Behaviours towards Immunisation Programmes: Vaccine Hesitancy during the COVID-19 Pandemic Era. Int. J. Environ. Res. Public Health.

[31] Arghittu A., Dettori M., Azara A., Gentili D., Serra A., Contu B., Castiglia P. (2020). Flu Vaccination Attitudes, Behaviours, and Knowledge among Health Workers. Int. J. Environ. Res. Public Health.

[32] Heine M., Hanekom S. (2023). Chronic Disease in Low-Resource Settings: Prevention and Management Throughout the Continuum of Care—A Call for Papers. Int. J. Environ. Res. Public Health.

[33] Asogwa O.A., Boateng D., Marzà-Florensa A., Peters S., Levitt N., van Olmen J., Klipstein-Grobusch K. (2022). Multimorbidity of non-communicable diseases in low-income and middle-income countries: A systematic review and meta-analysis. BMJ Open.

[34] Stephen C., McInnes S., Halcomb E. (2017). The feasibility and acceptability of nurse-led chronic disease management interventions in primary care: An integrative review. J. Adv. Nurs..

[35] Baysal G., Çiftci N., Dündar I., Büyükavcı M.A., Yağın F.H., Çamtosun E., Doğan D., Akıncı A. (2023). Chronic Disease Management of Children Followed with Type 1 Diabetes Mellitus. J. Clin. Res. Pediatr. Endocrinol..

[36] Thomas-Purcell K., Davenport R., Ayala V., Purcell D., Ownby R.L. (2023). Chronic Disease Self-Management of Post-Acute Sequelae of COVID-19 Among Older Adults: A Mixed-Methods Analysis. Clin. Interv. Aging.

[37] Oni T., Unwin N. (2015). Why the communicable/non-communicable disease dichotomy is problematic for public health control strategies: Implications of multimorbidity for health systems in an era of health transition. Int. Health.

[38] World Health Organization Primary Health Care. https://www.who.int/news-room/fact-sheets/detail/primary-health-care.

[39] Alotaibi H.M., Alruwaili Z.M., Dilli A.A., Altaleb A.A., Asiri M.M., Alwadani O.J., Alshaalan Z.M., Dar U.-F. (2023). Assessment of Primary Care Physicians’ Expertise of Common Dermatological Conditions in the Jouf Region, Saudi Arabia: A Mixed Methods Study. Healthcare.

[40] Sirirak T., Sangsupawanich P., Wongpakaran N., Srisintorn W. (2022). The Geriatric Depression Scale Predicts Glycemic Control in Older Adult with Type 2 Diabetes Mellitus: A Longitudinal Study. Healthcare.

[41] Arghittu A., Dettori M., Deriu G.M., Soddu S., Manca P.C., Carboni A.A., Collu I., Palmieri A., Deiana G., Azara A. (2022). Controlling Infectious Risk in Transfusion: Assessing the Effectiveness of Skin Disinfection in Blood Donors. Healthcare.

[42] Deiana G., Arghittu A., Gentili D., Dettori M., Palmieri A., Masia M.D., Azara A., Castiglia P. (2022). Impact of the COVID-19 Pandemic on the Prevalence of HAIs and the Use of Antibiotics in an Italian University Hospital. Healthcare.

[43] Stefanelli P., Bellino S., Fiore S., Fontana S., Amato C., Buttinelli G. (2019). Regional Reference Centres of the National Surveillance System for Acute flaccid paralysis. Hospital discharges-based search of acute flaccid paralysis cases 2007–2016 in Italy and comparison with the National Surveillance System for monitoring the risk of polio reintroduction. BMC Public Health.

[44] Balabanova D., Mills A., Conteh L., Akkazieva B., Banteyerga H., Dash U., Gilson L., Harmer A., Ibraimova A., Islam Z. (2013). Good Health at Low Cost 25 years on: Lessons for the future of health systems strengthening. Lancet.

[45] Physician Workforce Projections the Complexities of Physician Supply and Demand: Projections from 2019 to 2034. https://www.aamc.org/data-reports/workforce/data/complexities-physician-supply-and-demand-projections-2019-2034.

[46] Organisation for Economic Cooperation and Development Health at a Glance: Europe 2022: State of Health in the EU Cycle. https://www.oecd-ilibrary.org/social-issues-migration-health/health-at-a-glance-europe-2022_507433b0-en.

[47] Tediosi F., Lönnroth K., Pablos-Méndez A., Raviglione M. (2020). Build back stronger universal health coverage systems after the COVID-19 pandemic: The need for better governance and linkage with universal social protection. BMJ Glob. Health.

[48] Choksomngam Y., Jiraporncharoen W., Pinyopornpanish K., Narkpongphun A., Ongprasert K., Angkurawaranon C. (2022). Associations between Family Functioning and Symptoms of Attention-Deficit Hyperactivity Disorder (ADHD): A Cross-Sectional Study. Healthcare.

[49] Herbaux C., Dupré A., Rénier W., Gabellier L., Chazard E., Lambert P., Sobanski V., Gosset D., Lacroix D., Truffert P. (2022). Formative Assessment of Diagnostic Testing in Family Medicine with Comprehensive MCQ Followed by Certainty-Based Mark. Healthcare.

[50] Jaber D., Basheer H.A., Elsalem L., Dweib M., Khadra M., Abduljabbar R., Ghazwi R., Alhamad H. (2022). Prevalence and Predictive Factors of Masked Depression and Anxiety among Jordanian and Palestinian Couples: A Cross-Sectional Study. Healthcare.

[51] Park C.-H., Yoon K.J., Lee Y.-T., Jin S.M., Lee S.H., Kim T.H. (2022). Impact of Low Skeletal Muscle Mass and Obesity on Hearing Loss in Asymptomatic Individuals: A Population-Based Study. Healthcare.

[52] Alruwaili B.F., Thirunavukkarasu A., Alsaidan A.A., Al-Ruwaili A.M., Alanazi R.B.S., Alruwaili A.M.B., Alruwaili A.O., Altaymani A.M. (2022). Knowledge, Attitude, and Practice towards Evidence-Based Medicine among Northern Saudi Primary Care Physicians: A Cross-Sectional Study. Healthcare.

[53] Tokovska M., Šolcová J. (2022). The Burden of Next-of-Kin Carers of Dementia Sufferers in the Home Environment. Healthcare.

[54] Luvira V., Nonjui P., Butsathon N., Deenok P., Aunruean W. (2023). Family Background Issues as Predictors of Mental Health Problems for University Students. Healthcare.

[55] Schrimpf A., Bleckwenn M., Braesigk A. (2023). COVID-19 Continues to Burden General Practitioners: Impact on Workload, Provision of Care, and Intention to Leave. Healthcare.

[56] Frèche B., Salvan J., Roch M.C., Guerin A., Poupin E., Pichon M., Burucoa C. (2023). Diagnostic and Therapeutic Management of *Helicobacter pylori* Infection in Primary Care: Perspective of Application in France and Narrative Review of the Literature. Healthcare.

[57] Rivera L.R., Martínez M.S., Shigematsu L.M.R., García J.A.G., Corrales F.A., Toledano-Toledano F., Tapia A.J., Orozco D.I.T., García C.I.A. (2023). Violence against Women during the COVID-19 Pandemic in Mexico. Healthcare.

[58] Kosmalski M., Deska K., Bąk B., Różycka-Kosmalska M., Pietras T. (2023). Pharmacological Support for the Treatment of Obesity—Present and Future. Healthcare.

[59] Giannetta N., Katigri M.R., Azadboni T.T., Caruso R., Liquori G., Dionisi S., De Leo A., Di Simone E., Rocco G., Stievano A. (2023). Knowledge, Attitude, and Behaviour with Regard to Medication Errors in Intravenous Therapy: A Cross-Cultural Pilot Study. Healthcare.

[60] Hamdan N., Yaacob L.H., Idris N.S., Majid M.S.A. (2023). Primary Care Physicians’ Knowledge and Attitudes Regarding Palliative Care in Northeast Malaysia. Healthcare.

[61] Abouelkheir M., Taha A.E., Thirunavukkarasu A., Alkhamsan W.S.S., Almutairi F.K.S., Alanazi A.A.A., Alruwaili A.L.M., Alriwely N.S. (2023). Knowledge and Attitude towards Clinical Trials among General Population of Northern Saudi Arabia during COVID-19 Era: A Cross-Sectional Study. Healthcare.

[62] Wiedermann C.J. (2023). Revitalizing General Practice: The Critical Role of Medical Schools in Addressing the Primary Care Physician Shortage. Healthcare.

[63] Wang Y.-Y., Huang X.-C., Yuan J., Wu Y.-B. (2023). Exploring the Link between Family Health and Health Literacy among College Students: The Mediating Role of Psychological Resilience. Healthcare.

